# On the Elimination of Infections Related to Oncogenic Human Papillomavirus: An Approach Using a Computational Network Model

**DOI:** 10.3390/v13050906

**Published:** 2021-05-13

**Authors:** Cintia Muñoz-Quiles, Javier Díez-Domingo, Luis Acedo, Víctor Sánchez-Alonso, Rafael J. Villanueva

**Affiliations:** 1 Vaccine Research Area, FISABIO-Public Health, Avenida de Cataluña, 21, 46020 Valencia, Spain; cinquiles@gmail.com (C.M.-Q.); jdiezdomingo@gmail.com (J.D.-D.); 2 Department of Mathematics, Centro Universitario de Plasencia, University of Extremadura, 10600 Plasencia, Spain; acedo@unex.es; 3 Instituto Universitario de Matemática Multidisciplinar, 8G Building, 2nd Floor, Camino de Vera, Universitat Politècnica de Valéncia, 46022 Valencia, Spain; vicsana@doctor.upv.es

**Keywords:** human papillomavirus virus, cervical cancer, random network model, vaccination programs, oncogenic HPV elimination

## Abstract

Cervical cancer is the fourth most common malignancy in women worldwide, although it is preventable with prophylactic HPV vaccination. HPV transmission-dynamic models can predict the potential for the global elimination of cervical cancer. The random network model is a new approach that allows individuals to be followed, and to implement a given vaccination policy according to their clinical records. We developed an HPV transmission-dynamic model on a lifetime sexual partners network based on individual contacts, also accounting for the sexual behavior of men who have sex with men (MSM). We analyzed the decline in the prevalence of HPV infection in a scenario of 75% and 90% coverage for both sexes. An important herd immunity effect for men and women was observed in the heterosexual network, even with 75% coverage. However, HPV infections are persistent in the MSM population, with sustained circulation of the virus among unvaccinated individuals. Coverage around 75% of both sexes would be necessary to eliminate HPV-related conditions in women within five decades. Nevertheless, the variation in the decline in infection in the long term between a vaccination coverage of 75% and 90% is relatively small, suggesting that reaching coverage of around 70–75% in the heterosexual network may be enough to confer high protection. Nevertheless, HPV elimination may be achieved if men’s coverage is strictly controlled. This accurate representation of HPV transmission demonstrates the need to maintain high HPV vaccination coverage, especially in men, for whom the cost-effectiveness of vaccination is questioned.

## 1. Introduction

Cervical cancer is the fourth most common malignancy in women worldwide, but through vaccination with the human papillomavirus (HPV) vaccine and screening with cervical smear tests, it is a preventable disease [1]. Mathematical models have been developed in recent years in response to the global call to action towards the elimination of HPV-related cervical cancer made by the World Health Organization’s General Director in May 2018. In order to understand whether achieving elimination is possible, academic groups commissioned by the Secretariat have modeled the epidemiology of HPV infection and cancer, and the efficacy of HPV vaccination strategies, screening, and treatment [2].

Virtually all mathematical models have predicted that vaccinating girls against HPV is strongly cost-effective [3], but the same cannot be concluded for vaccinating boys [4,5]. Brisson et al. [6], in a systematic review and meta-analysis of model predictions, concluded that the elimination of oncogenic HPV genotypes included in the quadrivalent vaccine will be reached after 80 years of vaccinating boys and girls with a coverage of 80%. Simms et al. [7], using a dynamic mathematical model, postulated that 80–100% vaccine coverage with a nine-valent vaccine in girls only combined with HPV-based screening twice per lifetime with 70% uptake could reduce the worldwide mean age-adjusted cervical cancer incidence to less than 4 per 100,000 women in the years between 2020 and 2080. Brisson and Drolet [8] highlighted some limitations of these models, notably that they are unable to capture the differences in sexual behavior between countries and regions, and also fail to include high-risk groups with a significant role in the transmission dynamics of HPV. For instance, men who have sex with men (MSM) have high HPV prevalence and would not benefit from the herd immunity effect conferred by immunizing women. These considerations seem to be very difficult to model using classical dynamic mathematical models.

The classical HPV transmission-dynamic models used so far lack the flexibility to monitor the evolution of diseases in which the main route of transmission is contact among individuals. These continuous models cannot distinguish among individuals and do not track their clinical history using labels such as age, sex, previous illnesses, or infections, among others.

Furthermore, the previously mentioned models, as well as others [9,10], are based on systems of differential equations that assume there is homogeneous mixing in the population [11,12], i.e., any infectious individual can contact and infect any susceptible individual. This hypothesis may be reasonable in infectious diseases in general but does not seem appropriate in sexually transmitted diseases (STDs).

An approach using an agent-based model using the simulation model STDSIM to study the HPV dynamics can be seen in [13]. STDSIM was introduced in [14] with the aim of supporting decisions to control sexually transmitted diseases.

We previously developed an HPV transmission dynamics model on a lifetime sexual partners (LSP) network that uses individual contacts to monitor the evolution of HPV infections; this allows a more detailed and realistic approach than traditional continuous models [15]. This model was able to reproduce the initial results of the Australian vaccination campaign in terms of the rapid decline in the incidence of genital warts even after just two years of vaccination in girls [16]. This result was completely unexpected from the perspective of continuous models and pointed to an enhanced herd immunity effect in men and women as a result of vaccination in women.

**Figure 1:**
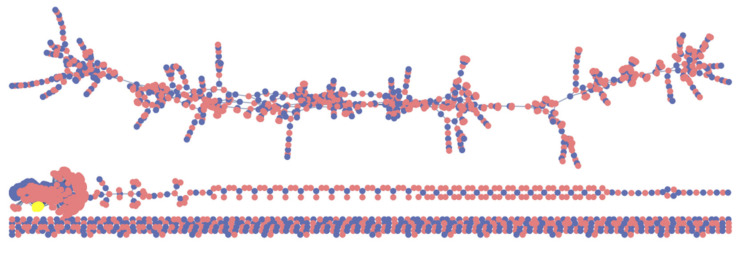
Lifetime sexual partners (LSP) network with 2500 nodes. Pink dots are women, blue dots heterosexual men and yellow dots men who have sex with men (MSM), zoomed in in Figure 2. The lower part corresponds to smaller sub-networks.

Unlike most of the models, this network model considered the MSM population, which accounts for about 4% of the whole population in Spain [17]. This sub-population is indeed a sub-network characterized by a larger number of sexual contacts [18] and also sporadic sexual contact with women. Figure 1 shows an LSP network with 2500 nodes.

**Figure 2:**
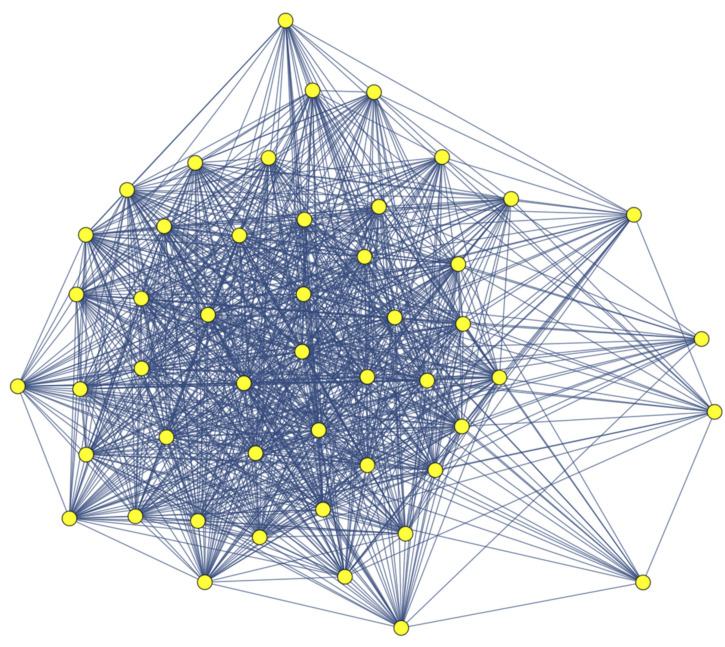
Men who have sex with men (MSM) sub-network for the LSP network in Figure 1. Any infected individual can infect any other directly or in a very short transmission path. This situation may be more reasonably modeled under the hypothesis of homogeneous mixing.

Figure 2 represents the MSM sub-network of sexual partners of the LSP network shown in Figure 1, where individuals may become infected through short transmission paths. As the transmission dynamics in the MSM sub-network may be reasonably modeled by classical models, we should expect that the herd immunity effect will be difficult to see until high vaccination coverage has been reached [11,12].

In this paper, we used a computational network model to predict the best strategy for the potential elimination of oncogenic HPV-related cancers by analyzing the decline in the prevalence of oncogenic HPV genotypes in two vaccination scenarios with the nine-valent vaccine, corresponding to 75% and 90% coverage for both sexes.

## 2. Materials and Methods

### 2.1. Computational Network Model Building

A network consists of nodes, representing individuals, and links or bonds between pairs of individuals, representing contacts (effective or not) for disease transmission. On a network, the evolution of the transmission of an infectious disease over time can be simulated using computer programs. In network models, we can follow any individual and implement a given vaccination policy by selecting them according to their clinical records, which is more difficult in classical models.

The main problem with building a realistic random network for STDs is the lack of data. While there have been some attempts to ascertain the structure of networks of sexual contacts of moderate size, they are insufficient in the context of large-scale transmission. The network of Bearman et al. [19] was fed with a dataset of 800 adolescents in a mid-sized town in the United States. However, although it uncovered a cycle structure for these networks, it cannot easily be extrapolated to larger networks. In the absence of enough experimental data to support a particular type of network, we assumed that a large-scale network of sexual contacts could be similar to a random network in which individuals are characterized by a given number of contacts or bonds assigned randomly to other individuals. In fact, the assignment is not completely random, but takes into account the similarity of individuals, as explained below.

Data on sexual behavior specific to Spain were collected from the Health and Sexual Habits Survey 2003, obtaining statistical information about the number of sexual partners until a certain age for both men and women [17]. The data are classified into age groups, as shown in Table 1.

For each sex and age group, Table 1 indicates the proportion of individuals with a given number of LSPs from 0 to 10 or more. The LSP refers to the total number of sexual partners of an individual until the time at which the survey was conducted.

We developed an algorithm to build large networks (from 100,000 up to 750,000 nodes) and an assignment algorithm to match couples of sexual partners. This algorithm should assign partners in such a way that the data in Table 1 are satisfied. Furthermore, the assignment should reproduce the way the people pair. In [20], the author found similarities in behavior and age in the couples. That is, people with similar age and similar behavior are more likely to be connected. These similarities are usually defined as assortatvity. Further technical details can be found in previous publications [15,21].

This gives us a static picture of the network, but it is also important to take its evolution into account in order to obtain the most realistic simulations possible. To do so, we assumed a constant population and adapt the sexual behavior when the node grew and changed to a new age group. As the individuals got older, we updated the number of bonds in such a way that the statistics in Table 1 were preserved.

Moreover, the MSM population was also taken into account. In our model, this population accounted for 3.88% of the whole population [17]. This sub-population was indeed a sub-network characterized by a higher average number of contacts [18] and also sporadic sexual contact with women, i.e., bisexual behavior (Díaz M., personal communication, Catalan Institute of Oncology, 2016). To our knowledge, data for bisexual contacts are not available. Thus, due to the high number of sexual partners of MSM, and following the principle of assortativity, we assumed that each MSM would have random contacts with a woman with a high number of LSPs (5 or more, to be precise).

Our model also takes into account infections by the high-risk HPV oncogenic genotypes 16/18/31/33/45/52/58. The nine-valent vaccine commercially available in Spain since 2017 protects against these [22], and we assumed permanent protection conferred by the vaccine. The evolution of spread of the infections was simulated with a discrete version of the SIS model, in which every node/individual may be susceptible or infected by the oncogenic genotypes mentioned above.

A set of probabilities was also needed and calibrated to take into account the transitions among these states:

- The global frequencies of sexual intercourse per age group and time. These frequencies may differ for each age group.
- The average recovery time from an oncogenic HPV infection.
- Two parameters determining if the oncogenic HPV infection is transmitted from a man or woman to his/her partner.

To calibrate the model, we used data retrieved from the CLEOPATRE study [23], in which the prevalence of different HPV genotypes in women was determined for a selected population in Spain. This calibration was performed probabilistically, returning 95% confidence intervals (CI) of the model parameter values and the model outputs. Details of the calibration can be found in ref. [21] and the calibrated parameters are shown in Table 2.

We used networks with a large number of nodes (100,000) jointly with the calibrated model parameter values to simulate the vaccination scenarios of interest with the nine-valent vaccine and the evolution of the oncogenic HPV infections over time.

### 2.2. Scenarios to Be Simulated

Using our computational model, we simulated the situation before vaccination. We considered the year when the vaccination program starts to be year 0. The program consists of vaccinating boys and girls with a given coverage in such a way that, when they turn 14 years old and enter into the system, they are already vaccinated. We then performed the simulation in order to study the dynamics of the oncogenic HPV over the next 100 years. Accordingly, we calculated the decline in the prevalence of oncogenic HPV in women, men, and MSM (MSM are also included in men) over the time measured as *D_t_* = 100 (1 − *P_t_*/*P*), where *D_t_* is the decline in oncogenic HPV in the time instant (month) *t*, *P_t_* is the prevalence of the oncogenic HPV in the time instant *t*, and *P* is the prevalence of oncogenic HPV just before the vaccination program starts.

Here, we considered the vaccination program with equal coverage for boys and girls of 75% and 90%. The decline in oncogenic HPV infection is an indicator of whether the related cancers can be eliminated. We can also estimate when levels of decline of 65%, 75%, 85%, and 95% are reached by women, men, and MSM.

## 3. Results

Table 3 shows the number of years needed from the start of the vaccination program to reach infection decline levels of 65%, 75%, 85%, and 95% for vaccine coverage of 75% and 90% in men and women. Note that men and women can reach levels of decline higher than the coverage percentages, which reflects the existence of a significant herd immunity effect. This effect does not appear in MSM decline. 

**Figure 3:**
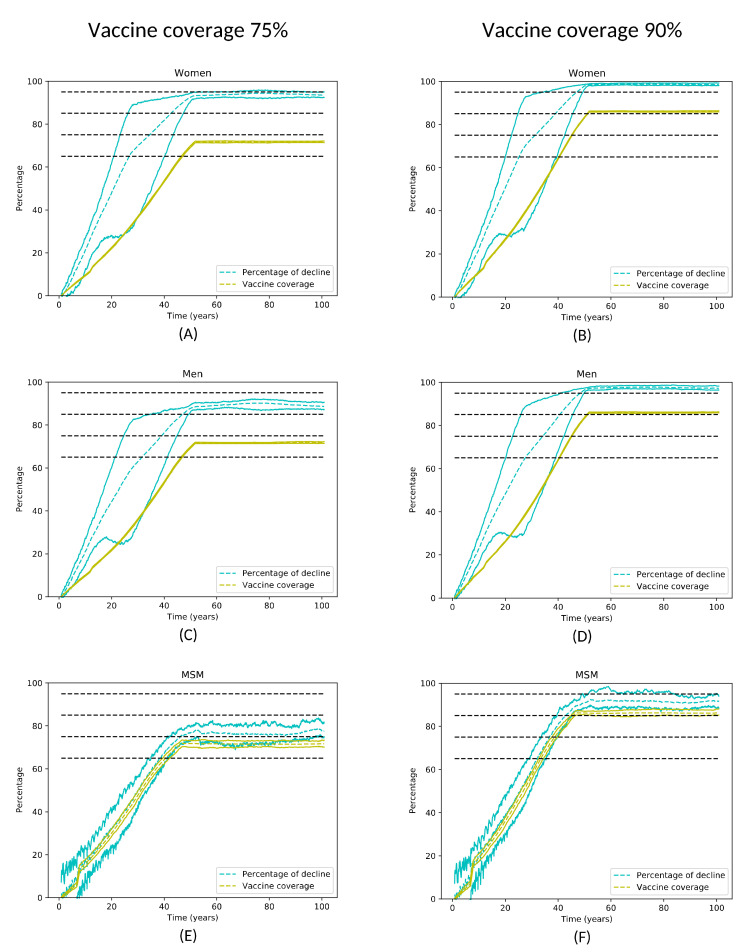
Decline in the number of infections caused by oncogenic HPV genotypes in women (**A**,**D**), men (**B**,**E**), and MSM (**C**,**F**) aged 14–64 years over the time since the start of vaccination, with 75% (**A**–**C**) and 90% (**D**–**F**) vaccine coverage. The vaccine coverage in each graph is given by the green curve and the percentage of decline in the incidence of infections is given by the cyan curve with 95% confidence intervals. Time represents the time since the beginning of vaccination in years. Dashed horizontal lines correspond to declines of 65%, 75%, 85%, and 95%.

Figure 3 shows graphs illustrating the decline in oncogenic HPV in 14- to 64-year-old women, men, and MSM with coverage for men and women of 75% and 90%. The difference between the percentage of vaccinated individuals (yellow lines) and the decline in HPV (cyan lines) is a measure of the herd immunity effect on each population. A sizeable herd immunity effect for women and the whole male population was observed, even with a moderate coverage of 75%. As we can see, the herd immunity effect on men and women is similar. The small differences are because men include MSM, even though the percentage of MSM is low with respect to the total number of men. Nevertheless, in MSM, the herd immunity is low and only visible in the long term, because of the high number of LSPs of MSM.

Furthermore, the highest values of decline are reached at around 50 years in all the graphs, i.e., after a complete generation has been vaccinated, since our model considers individuals aged 14–64 years old; accordingly, 50 years is a complete generation.

Nevertheless, Figure 3 also shows that the variation in the decline in the long term between the vaccination of 75% to 90% is small if we consider that we need a 15% increase in coverage. From year 50 to 100, the differences in the decline between coverage of 90% and 75% are: 6.3% to 10.1% in men; 3.3% to 6.4% in women; and 10.2% to 20% for MSM. The increase is fully reflected in the similar order of magnitude, around 15%, for MSM coverage only. In other words, looking at Table 3, with the increase in coverage from 75% to 90%, on average, levels of decline of 65%, 75% and 85% are reached 2–4 years earlier for women and 4–6 years earlier for men. For MSM, levels of decline of 65% and 75% are reached 6–9 years earlier, on average. This suggests that reaching coverage of around 70–75% is enough to confer high protection at the population level without significantly increasing the vaccination cost to achieve small increases in the decline in the long term. Nevertheless, the highest coverage possible would have to be maintained for MSM. Therefore, considering that sexual preferences of MSM appear after vaccination age [24], men’s coverage needs to be strictly fulfilled. This is consistent with the findings of Villanueva et al. [25], where a simulation showed that the vaccination of men (heterosexual men and MSM) provides a significantly higher protective effect on women than the protective effect on men if women are vaccinated.

## 4. Discussion

In November 2020, a global strategy to accelerate the elimination of cervical cancer was published by the World Health Organization [2]. Here, we attempted to estimate the timeline of possible HPV-related infection elimination through gender-neutral HPV vaccination, provided significant vaccination coverage is reached. We developed a computational random network model that simulates contacts between heterosexual males and females over time according to their age as they get older, and also takes into account a sub-network of MSM who have sporadic sexual contact with females. According to our simulations, in a scenario where 90% of both male and female populations are vaccinated, a period of 50 years would be necessary to achieve a 95% decline in the infection rate, which corresponds to vaccinating a whole generation. This finding is consistent with recent publications worldwide [6,7,8]. Nevertheless, the variation in infection decline in the long term between vaccination coverage of 75% and 90% is relatively small, suggesting that reaching a coverage of around 70–75% in the heterosexual network may be enough to confer high protection at population level, thanks to herd immunity effects arising in both the male and female heterosexual population. This result is in line with the conclusions of Lehtinen et al. [26]. A recent study carried out in the Netherlands, a country with relatively moderate girls-only bivalent vaccine uptake, also showed a herd effect between heterosexual men and unvaccinated women since the vaccination program began [27]. Furthermore, in Spain, anogenital warts incidence at the late teens declined by 61% after 8 years of systematic HPV vaccination of 11-year old girls [28].

After Australia implemented a girls-only vaccination program, it saw a rapid 93% decline (from 11.5% in 2007 to 0.85% in 2011) in cases of genital warts diagnosed in females aged <21 years and an 82% decline in cases in unvaccinated heterosexual males aged <21 years (from 12.1% in 2007 to 2.2% in 2011) with a vaccine coverage of 73%. Estimates for HPV-related cancers in Australia have predicted that an annual incidence of <4 cases per 100,000 women will be achieved by 2034, meaning elimination if vaccine coverage among girls and HPV-screening are maintained [29]. However, no significant decline was seen in anogenital warts in MSM [24]. In fact, our model, with its accurate replication of the herd immunity effect, also highlights the fact that the MSM population is associated with a comparatively much smaller herd immunity effect in the short term. Unprotected MSM may maintain circulation of the virus due to a higher number of contacts per individual. In this respect, in a recent paper, Bogaards et al. [30] proposed and studied different strategies to encourage MSM vaccine uptake.

Thus, once a successful HPV-vaccination program for girls has been established, targeted HPV vaccination for MSM appears to be the next priority for the prevention of HPV-related malignancies [31]. Pollock et al. [32] recently studied the HPV vaccine uptake in MSM in Scotland in an opportunistic program, with vaccination being offered in the sexual health clinic setting. They concluded that the first-dose uptake was moderately high but suggested that gender-neutral immunization would be preferable to MSM-targeted vaccination, reducing stigma and inequality. Furthermore, another study found that a program targeted to young MSM only would not be effective before or shortly after their sexual debut, as most MSM disclose their sexual preference in their early twenties [24]. In fact, a recent real-world study carried out in the United States, where MSM HPV-vaccination has been recommended since 2011, proved that MSM vaccination is effective and that its effectiveness is higher when the vaccine is administered before 18 years of age [33]. Another study conducted in France, where the HPV-vaccine has been reimbursed for MSM under 27 years of age since 2017, saw an increase in MSM immunization since then [34]. It should also be considered that any increase in the MSM population over time would make it slightly more difficult to eliminate HPV-related infections. Thus, although previous studies determined that vaccination for heterosexual men would only be cost-effective at a lower price than the current one, gender-neutral vaccination before sexual debut would prevent HPV persistence in the MSM subpopulation with sporadic sexual contact with women [24], and HPV elimination may be reached if men’s coverage is strictly controlled. Indeed, a recent cost-effectiveness study published for France suggested that, over a hundred-year time horizon, gender-neutral vaccination could be considered cost-effective compared with girls-only vaccination [35]. Furthermore, the European Centre for Disease Prevention and Control, in a report [36], concluded that the gender-neutral vaccination is cost-effective if all HPV-related diseases are considered.

### Strengths and Limitations

An important strength of our model is its ability to reflect the evolution of sexual behavior depending on the age of individuals. As a node grows and changes to a new age group, the model updates the number of bonds, preserving the statistics from the literature. On the other hand, as with all computational models, the one we develop depends on the data used to feed it. Thus, it is influenced by possible biases in the data in terms of sexual behavior [17,18] and the source data used to calibrate it [23]. The lack of specific data on the sexual behavior of the population prevents us from checking the reliability of the network structure. Unfortunately, the available data are quite old, and the sexual behavior of the population may have changed significantly. With respect to the behavioral parameters for MSM, these are obtained from a relatively young population that was, therefore, more sexually active. Thus, we may have overestimated the required vaccination threshold for a 90% reduction in infection.

However, we performed a sensitivity analysis considering: (a) an increase in the percentage of MSM up to 10%; (b) a twofold increase in the frequency of intercourse per age group; (c) an increase in the LSP, where the distribution percentages in Table 1 were maintained but changing the number of LSPs. Thus, the proportion of people with only 1 LSP in the simulation had 2; people with 2 LSPs had 4; people with 3–4 LSPs had 6–8; people with 5–9 LSPs had 9–13; and the people with 10 or more LSPs had 14 or more. The number of LSPs of MSM was also increased fourfold. The sensitivity analysis returned very similar results for the decline in oncogenic HPV infections, which indicates that the model is quite resilient with respect to large size modifications. This sensitivity analysis was published in Acedo et al. [37].

In our model, we do not consider geographical and societal differences that might influence the sexual behavior and vaccine uptake. Moreover, the reduction in oncogenic HPV incidence/prevalence does not necessarily correspond to reductions in HPV-related cancers of the same magnitude as vaccination will disrupt the infection dynamics of HPV even for those who are still infected.

## 5. Conclusions

Our results suggest that health equity for MSM compared to heterosexuals for HPV infection will only be achieved with a gender-neutral vaccination program. HPV infections are resilient in the MSM population, with persistent circulation of HPV among unvaccinated individuals. This accurate representation of HPV transmission through a network model considering MSM sexual behavior shows the need to maintain the highest HPV vaccination coverage, especially in men, when strategies aimed at elimination of cervical cancer are under scrutiny. Therefore, the elimination of the cancers related to oncogenic HPV genotypes is possible with sufficiently high coverage, vaccinating men and women, and closely monitoring coverage in men to keep it as high as possible in order to have special care with the vaccination of MSM.

## Figures and Tables

**Table 1:** Proportion of males and females per number of lifetime sexual partners (LSP) per age group. Note that the sum of the rows is 1.

Males						
**Age**	**0 LSP**	**1 LSP**	**2 LSP**	**3−4 LSP**	**5−9 LSP**	**10 or more LSP**
14–29	0.107	0.207	0.131	0.225	0.168	0.162
30–39	0.027	0.225	0.128	0.21	0.17	0.24
40–65	0.019	0.268	0.14	0.193	0.163	0.217
**Females**						
**Age**	**0 LSP**	**1 LSP**	**2 LSP**	**3−4 LSP**	**5−9 LSP**	**10 or more LSP**
14–29	0.138	0.43	0.186	0.158	0.056	0.032
30–39	0.029	0.501	0.168	0.177	0.077	0.048
40–65	0.017	0.652	0.138	0.118	0.039	0.036

**Table 2:** Calibrated model parameters. Details can be found in ref. [21].

Model Parameter	Mean	95% CI
Average LSP men	8.63	(7.15, 9.86)
Average time clearing oncogenic HPV (years)	1.08	(0.88, 1.19)
Probability a woman transmits oncogenic HPV	0.81	(0.68, 0.95)
Probability a man transmits oncogenic HPV	0.91	(0.74, 0.97)
Frequency, 14–17 years old	0.1098	(0.0485, 0.1542)
Frequency, 18–29 years old	0.0776	(0.0568, 0.0981)
Frequency, 30–39 years old	0.0620	(0.0024, 0.0935)
Frequency, 40–65 years old	0.0190	(0.0046, 0.0553)

**Table 3:** Table showing the year after the vaccination program starts in which the decline of oncogenic HPV reaches 65%, 75%, 85%, and 95% when vaccinating boys and girls with 75% and 90% coverage. MSM are included in men, and are also considered separately. The symbol “−” means that this percentage of decline was not reached during the simulation period.

Vaccination of Boys and Girls with 75% Coverage			
**Decline**	**Women**	**Men**	**MSM**
65%	year 26 95% CI (20, 39)	year 31 95% CI (20, 40)	year 37 95% CI (34, 41)
75%	year 34 95% CI (22, 42)	year 38 95% CI (24, 44)	year 45 95% CI (41, 51)
85%	year 42 95% CI (25, 46)	year 46 95% CI (34, 48)	year − 95% CI (–, –)
95%	year – 95% CI (52, –)	year – 95% CI (–, –)	year – 95% CI (–, –)
**Vaccination of Boys and Girls with 90% Coverage**			
**Decline**	**Women**	**Men**	**MSM**
65%	year 24 95% CI (19, 38)	year 27 95% CI (19, 38)	year 31 95% CI (28, 34)
75%	year 30 95% CI (21, 42)	year 33 95% CI (22, 40)	year 36 95% CI (33, 39)
85%	year 38 95% CI (24, 44)	year 40 95% CI (25, 44)	year 42 95% CI (39, 44)
95%	year 46 95% CI (34, 48)	year 47 95% CI (40, 49)	year – 95% CI (48, –)

## Data Availability

Data used in this work are those of the tables and the cited references and are publicly available.
